# Rapid left ventricular function MRI with an accelerated real-time-based spiral acquisition

**DOI:** 10.1186/1532-429X-18-S1-P326

**Published:** 2016-01-27

**Authors:** R Reeve Ingle, Justin J Konkle, Nii O Addy, Galen D Reed, Michelle M Nystrom, Kenneth O Johnson, William R Overall, Juan M Santos, Bob S Hu

**Affiliations:** HeartVista, Inc., Menlo Park, CA USA

## Background

Breath-held (BH) cardiac cine MRI is a widely used technique for the assessment of cardiac left ventricular (LV) function. Image quality can be severely compromised in subjects who cannot perform the required breath holds or in subjects with arrhythmia. Additionally, the series of breath holds required for full ventricular coverage leads to prolonged exam times. These drawbacks can be mitigated with real-time imaging approaches, but the limited spatial resolution of many real-time techniques can limit their utility for functional assessment. In this work, we develop a real-time-based multi-slice steady-state free precession (SSFP) sequence that utilizes an accelerated spiral acquisition and non-Cartesian SPIRiT reconstruction to increase spatial resolution.

## Methods

**Figure 1:**
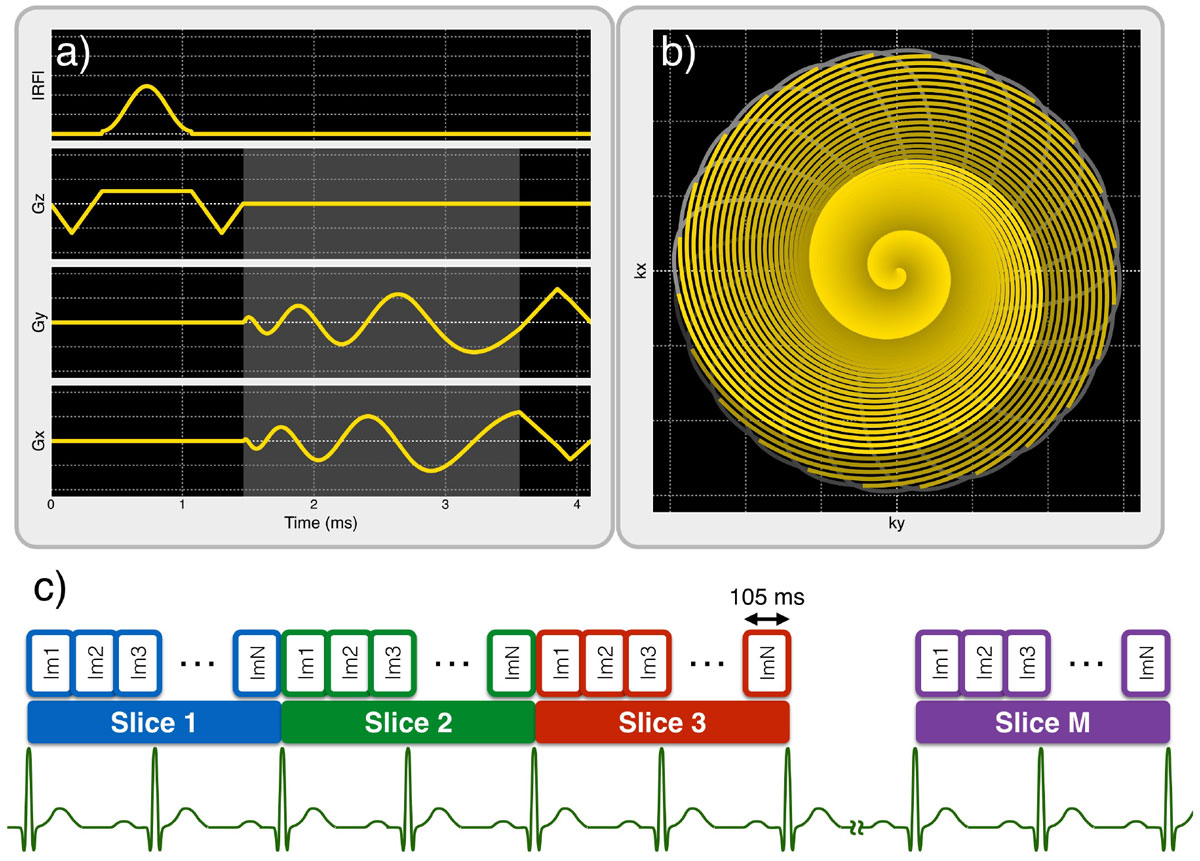
**Pulse sequence and acquisition strategy**. The pulse sequence diagram **(a)** shows the windowed-sinc excitation pulse and one spiral interleaf from the 24-interleave dual-density spiral readout. The *k*-space diagram **(b)** shows the *k*-space trajectories of all spiral interleaves. A 0.6-ms fully-sampled central region is used for SPIRiT kernel calibration. For each slice, the 24 spiral interleaves are acquired continuously for 2 R-R intervals **(c)**, and sliding window reconstruction is used to reconstruct 40 frames per slice location. The slice location is incremented every 2 R-R intervals until the desired volume has been imaged.

A 24-interleave dual-density spiral readout (4.5x-accelerated outer region) is repeatedly acquired for 2 R-R intervals per slice location using a 2D SSFP pulse sequence (Figure 1a-1b). The slice location is advanced every 2 R-R intervals until all slice locations have been acquired (Figure 1c). Pulse sequence design, acquisition, and image reconstruction were implemented using the RTHawk Research platform (HeartVista, Menlo Park, CA) and a GE 1.5 T TwinSpeed scanner. Five healthy subjects were scanned using the proposed real-time-based spiral sequence (spatial res. = 1.7 × 1.7 mm^2^, temporal res. = 105 ms, TR = 4.4 ms) and a conventional BH Cartesian cine sequence (spatial res. = 1.6 × 2.0 mm^2^, temporal res. = 45 ms, TR = 3.8 ms, 1.6x parallel acceleration). Nine short-axis slice locations covering the LV were imaged with both sequences (flip = 60°, FOV = 32 × 32 cm^2^, slice thickness = 8 mm). Cine breath holds were 11 heartbeats per slice for a total of 99 heartbeats, while the proposed technique acquired all slices during a single 18-heartbeat breath hold. Stroke volume (SV) and ejection fraction (EF) measurements were compared for the two techniques.

## Results

**Figure 2:**
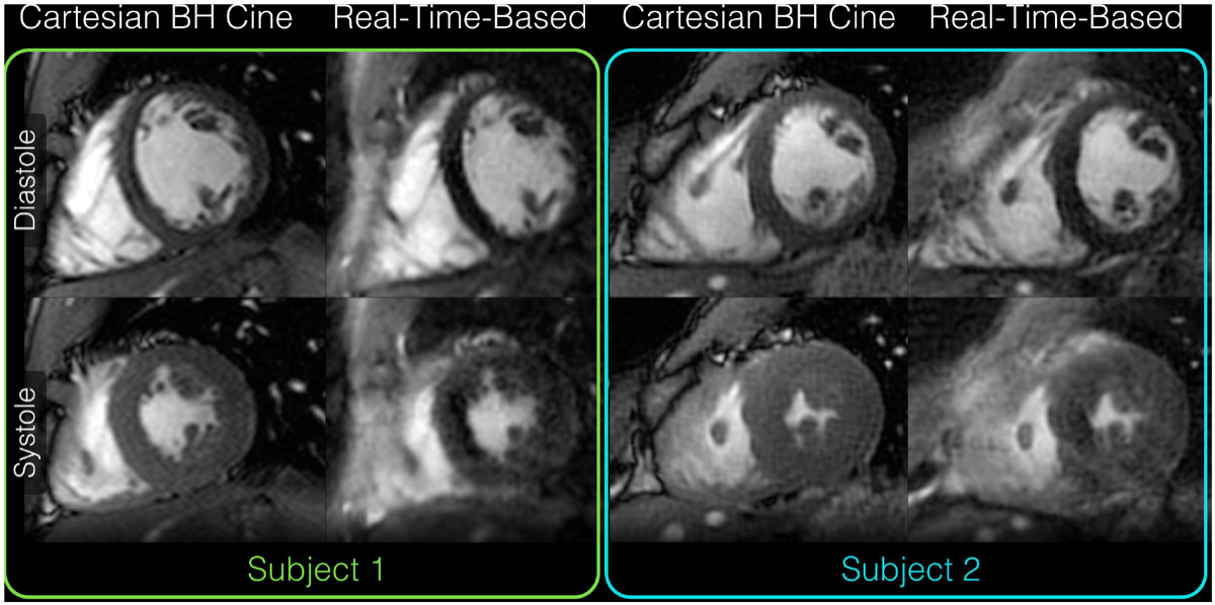
**Diastolic and systolic images are shown from two subject scans comparing the proposed real-time-based spiral acquisition with a conventional breath-held Cartesian cine acquisition**. To minimize slice location discrepancies between the two techniques, all slices from the real-time-based acquisition were acquired in a single 20-second breath hold. Cartesian cine images were acquired in 11 heartbeats per slice, 1.6 × 2.0 mm^2^ spatial resolution, and 45 ms temporal resolution. Real-time-based spiral images were acquired in 2 heartbeats per slice, 1.7 × 1.7 mm^2^ spatial resolution, and 105 ms temporal resolution.

Figure 2 compares conventional cine images and real-time-based spiral images from two subjects. Image quality is comparable between the two approaches. The proposed technique has higher flow sensitivity, yielding some artifacts over the myocardium in systolic images (Figure 2). For all subjects, the mean differences in SV (0.4 ± 4.6 mL, *p* = 0.9) and EF (-0.8 ± 3.2%, *p* = 0.6) between the proposed and conventional approaches were small and not statistically significant.

## Conclusions

A rapid, real-time-based multi-slice SSFP pulse sequence has been developed using an accelerated spiral acquisition to achieve comparable spatial resolution as conventional BH cine imaging in a fraction of the scan time. Preliminary in-vivo comparisons of the proposed technique to Cartesian cine SSFP showed no significant differences in SV or EF and similar image quality between the two techniques. Future work will include a larger patient study to assess robustness to arrhythmias and poor breath holding and to further compare cardiac functional measurements.

